# Ultralong‐Range Polariton‐Assisted Energy Transfer in Organic Microcavities

**DOI:** 10.1002/anie.202105442

**Published:** 2021-06-18

**Authors:** Kyriacos Georgiou, Rahul Jayaprakash, Andreas Othonos, David G. Lidzey

**Affiliations:** ^1^ Department of Physics and Astronomy University of Sheffield, Hicks Building Hounsfield Road Sheffield S3 7RH UK; ^2^ Department of Physics University of Cyprus P.O. Box 20537 Nicosia 1678 Cyprus

**Keywords:** energy transfer, J-aggregates, microcavity, organic semiconductor, polaritons

## Abstract

Non‐radiative energy transfer between spatially‐separated molecules in a microcavity can occur when an excitonic state on both molecules are strongly‐coupled to the same optical mode, forming so‐called “hybrid” polaritons. Such energy transfer has previously been explored when thin‐films of different molecules are relatively closely spaced (≈100 nm). In this manuscript, we explore strong‐coupled microcavities in which thin‐films of two J‐aggregated molecular dyes were separated by a spacer layer having a thickness of up to 2 μm. Here, strong light‐matter coupling and hybridisation between the excitonic transition is identified using white‐light reflectivity and photoluminescence emission. We use steady‐state spectroscopy to demonstrate polariton‐mediated energy transfer between such coupled states over “mesoscopic distances”, with this process being enhanced compared to non‐cavity control structures.

## Graphical abstract

Ultralong‐range polariton‐assisted energy transfer was achieved in a microcavity containing two J‐aggregate cyanine dyes. The two dyes were separated by a distance of up to 2 μm using a polymeric spacer layer. Here, it was confirmed that hybrid middle polariton (MP) states, composed of a mixture between the cavity photon modes and the different exciton states, act as a route for efficient ultralong‐range energy transfer to energetically lower‐lying states.

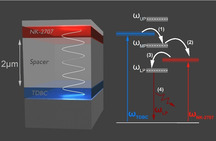

## Introduction

Exciton‐polaritons are bosonic quasi‐particles formed when the electronic excitations of matter strongly couple to a confined optical field.^[1]^ Strong coupling in organic‐semiconductor materials has attracted particular attention due to the high binding energy of Frenkel‐excitons that permits polariton effects to be explored at room temperature.^[2]^ This key property has allowed to organic‐exciton polaritons to be used as a platform to study fundamental physics and phenomena at room temperature,[^3^, ^4^, ^5^, ^6^, ^7^, ^8^] with a number of practical applications being proposed.^[9]^

By placing two (or more) different organic semiconductor materials in a planar Fabry–Perot microcavity, it has been shown that the excitonic states associated with the different materials can become mutually hybridised through simultaneous coupling to the same photonic mode.^[10]^ This hybridisation has been shown to result in energy transfer between the different molecular species via mixed “middle‐branch” polaritons,[^11^, ^12^] with these states effectively acting as a transfer‐route between different excitonic reservoirs. These effects have been observed even when such hybridised molecules are separated by more than 100 nm.^[13]^ Energy transfer between donor‐acceptor species, exciton harvesting and polariton propagation has also been reported in other structural configurations in which organic excitons are coupled to either surface plasmons or Bloch surface waves.[^14^, ^15^, ^16^] Vibron hybridisation effects have also been observed at room temperature, by coupling two molecular vibrational modes to the same IR electromagnetic field.[^17^, ^18^, ^19^] Other work has demonstrated hybridisation of excitons in a 2D material and an organic semiconductor, with the degree of hybridisation being tuneable via application of an electric field.^[20]^ We note that excitonic hybridisation through strong coupling may enable the development of new technologies, such as an electrically‐injected, room‐temperature polariton laser; here the concept of polariton condensation has been demonstrated in a hybrid‐semiconductor device,^[21]^ with recent work demonstrating the electrical generation of organic‐exciton polaritons in a coupled‐cavity device containing a series of inorganic quantum wells.^[22]^ In such coupled‐cavity geometry, it has been recently shown that the degree of coupling and energy transfer between magnetically doped quantum well excitons can be controlled through the application of a magnetic field.^[23]^

We note that most studies of exciton hybridisation have explored energy transfer between materials that are physically separated by around ≈100 nm. While such a length‐scale is comfortably greater than either the Förster transfer radius or the typical Frenkel‐exciton diffusion length, it is interesting to explore whether polaritonic energy transfer effects could occur over much longer distances as this may open new applications. We have therefore explored this idea and have constructed a series of microcavities containing two hybridised J‐aggregated cyanine dyes separated by a polymeric spacer layer of variable thickness. Using angle‐resolved white light reflectivity and photoluminescence (PL) measurements, we show that such microcavities operate in the strong coupling regime. By comparing PL emission from microcavities and non‐cavity control films, we demonstrate that there is a substantial redistribution of emission inside a microcavity to lower energy polaritonic states. In such cavities we separate the active layers by distances up to 2 μm, suggesting efficient energy transfer occurs even over “mesoscopic” distances. This process is verified by photoluminescence excitation (PLE) that confirms that energy transfer is mediated by hybrid middle polariton states that are composed of an admixture of different excitonic states. Such long‐range energy transfer may potentially be of relevance in advanced solar cells, or in microfluidic structures that seek to harness polariton effects to modify chemical reactions.[^24^, ^25^, ^26^]

## Results and Discussion

**Figure 1:**
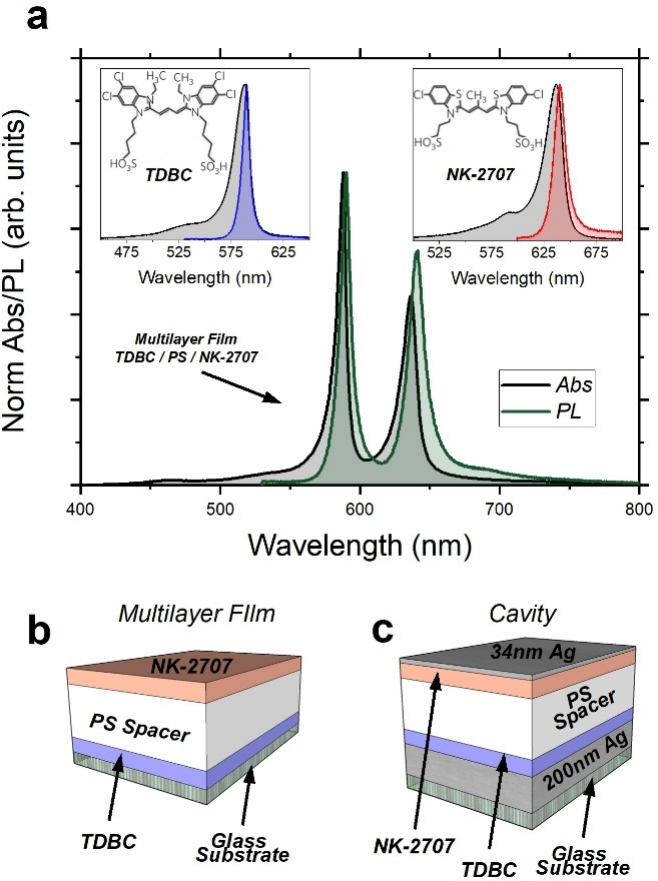
a) Absorption in black and PL emission in green (excited at 405 nm) from a TDBC/PS/NK‐2707 multilayer. The inset shows absorption and PL (excited at 405 nm) recorded from individual films of TDBC (black and blue) and NK‐2707 (black and red) along with their molecular structure. Parts (b) and (c) show schematics of (b) the multilayer film and (c) the microcavity structure explored in this study.

We have studied microcavities containing multilayer films composed of two different fluorescent cyanine dye J‐aggregates, namely 5,6‐dichloro‐2‐[[5,6‐dichloro‐1‐ethyl‐3‐(4‐sulphobutyl)‐benzimidazol‐2‐ylidene]‐propenyl]‐1‐ethyl‐3‐(4‐sulphobutyl)‐benzimidazolium hydroxide, sodium salt, inner salt (TDBC) and 5‐chloro‐2‐[3‐[5‐chloro‐3‐(3‐sulphopropyl‐2(3*H*)‐benzothiazolylidene]‐2‐methyl‐1‐propenyl]‐3‐(3‐sulphopropyl)‐benzothiazolium hydroxide, inner salt, compound with triethylamine (NK‐2707). Here, J‐aggregates are formed by self‐organisation when such dyes are dissolved in a polar solvent. J‐aggregates are often characterised by high oscillator strength and super‐radiant emission, together with relatively narrow red‐shifted absorption and PL emission as compared to their un‐aggregated monomers.^[27]^ Such optical properties make J‐aggregated dyes a popular system for use in strongly coupled structures and devices.[^11^, ^21^, ^28^] To process the dyes, they were first separately dissolved in a solution of DI water containing gelatine that acted as a matrix. On spin‐coating, the cyanine dyes underwent J‐aggregation as their local concentration increased. The inset of Figure 1 a plots the absorption and PL emission of control films of TDBC and NK‐2707 when deposited onto a quartz‐coated glass substrate, together with their molecular structure. As it can be seen, both J‐aggregated dyes are characterised by narrow absorption and PL features (with linewidths of the order of 10–20 nm) with the absorption and emission peaks of TDBC being centred around 588 nm and 590 nm, respectively, while absorption and emission of NK‐2707 is located at 637 nm and 641 nm, respectively. Both materials have a small Stokes shift between absorption and emission of the order of ≈10 meV.

Multilayer control films (Figure 1 b) were fabricated in which the TDBC and NK‐2707 layers were separated by a film of the optically transparent polymer polystyrene (PS). Here, it was possible to create a multilayer structure, as the different films had orthogonal solubility in the casting‐solvents water and toluene. To construct multilayer films, a TDBC film was first spin‐coated from gelatine/DI water solution. A PS spacer‐layer having a thickness of up to 2 μm was then spin‐cast onto the TDBC film from a toluene solution. Here, the negligible solubility of TDBC/gelatine in toluene permitted a stable structure to be fabricated. Finally, a NK‐2707/gelatine film was cast onto the PS surface by spin‐coating from a water‐based solution. Note that the concentration and thickness of the individual cyanine dyes in their matrix layers were adjusted such that the integrated PL emission intensity from the two materials was similar.

Figure 1 a shows absorption and PL emission of a control TDBC/PS/NK‐2707 multilayer deposited onto a quartz‐coated glass substrate (schematic of Figure 1 b). Two main peaks can be detected in absorption (energetically separated by ≈160 meV) that are associated with the absorption of TDBC and NK‐2707 layers. Here, the energy separation between the peaks is commensurate with the typical energetic Rabi splitting observed in strongly coupled organic microcavities[^2^, ^29^] and has allowed us to generate exciton hybridisation when such a multilayer is placed in a microcavity structure (see below). In green, we plot the multilayer PL emission following non‐resonant excitation at 405 nm using a CW diode laser. Again, two distinct well‐separated main peaks are observed; with a very weak shoulder being visible at around 700 nm that is most likely resulted from the 0–1 J‐aggregated emission of NK‐2707.^[30]^ Note that dipole–dipole interactions and Förster resonance energy transfer are strongly suppressed in such multilayer films as the spacer layer is much thicker than a typical Förster transfer radius.[^31^, ^32^, ^33^] This conclusion is further supported by femtosecond pump‐probe measurements on TDBC and TDBC/PS/NK‐2707 films that show very similar decay lifetimes detected at a wavelength of 588 nm, corresponding to the peak absorption wavelength of TDBC (see Figure S1 of the Supporting Information). We have also performed PLE measurements on multilayer films and have evidenced negligible energy transfer due to direct emission of photons by TDBC followed by their reabsorption by NK‐2707 (see Figure S2 of the Supporting Information).

We have fabricated two different microcavity structures containing multilayer TDBC/PS/NK‐2707 films placed between silver (Ag) mirrors, and—as we show below—both structures operate in the strong coupling regime. A schematic of the microcavities explored in this study is shown in Figure 1 c. To fabricate the microcavities, a 200 nm thick Ag mirror was first deposited onto a quartz‐coated glass substrate via thermal evaporation. The multilayer film comprising the two J‐aggregate layers separated by a PS spacer layer was then spin‐coated onto the silver mirror as described above. Finally, the cavity was completed by the thermal evaporation of a semi‐transparent 34 nm thick Ag mirror onto the top of the multilayer. Note that two different pairs of control non‐cavity films and strongly coupled cavities were fabricated. The first film–cavity pair comprised of a multilayer of TDBC/PS/NK‐2707 having a thickness of 141 nm/1160 nm/102 nm which we refer to (for the sake of simplicity) as Multilayer‐A and Cavity‐A. The second film–cavity pair consisted of a thicker PS spacer layer, but with both J‐aggregate layers having the same thickness as was used in Multilayer‐A and Cavity‐A. Here, the TDBC/PS/NK‐2707 layers had a thickness of 141 nm/2150 nm/102 nm, with the multilayer‐control and cavity structure referred to as Multilayer‐B and Cavity‐B.

**Figure 2:**
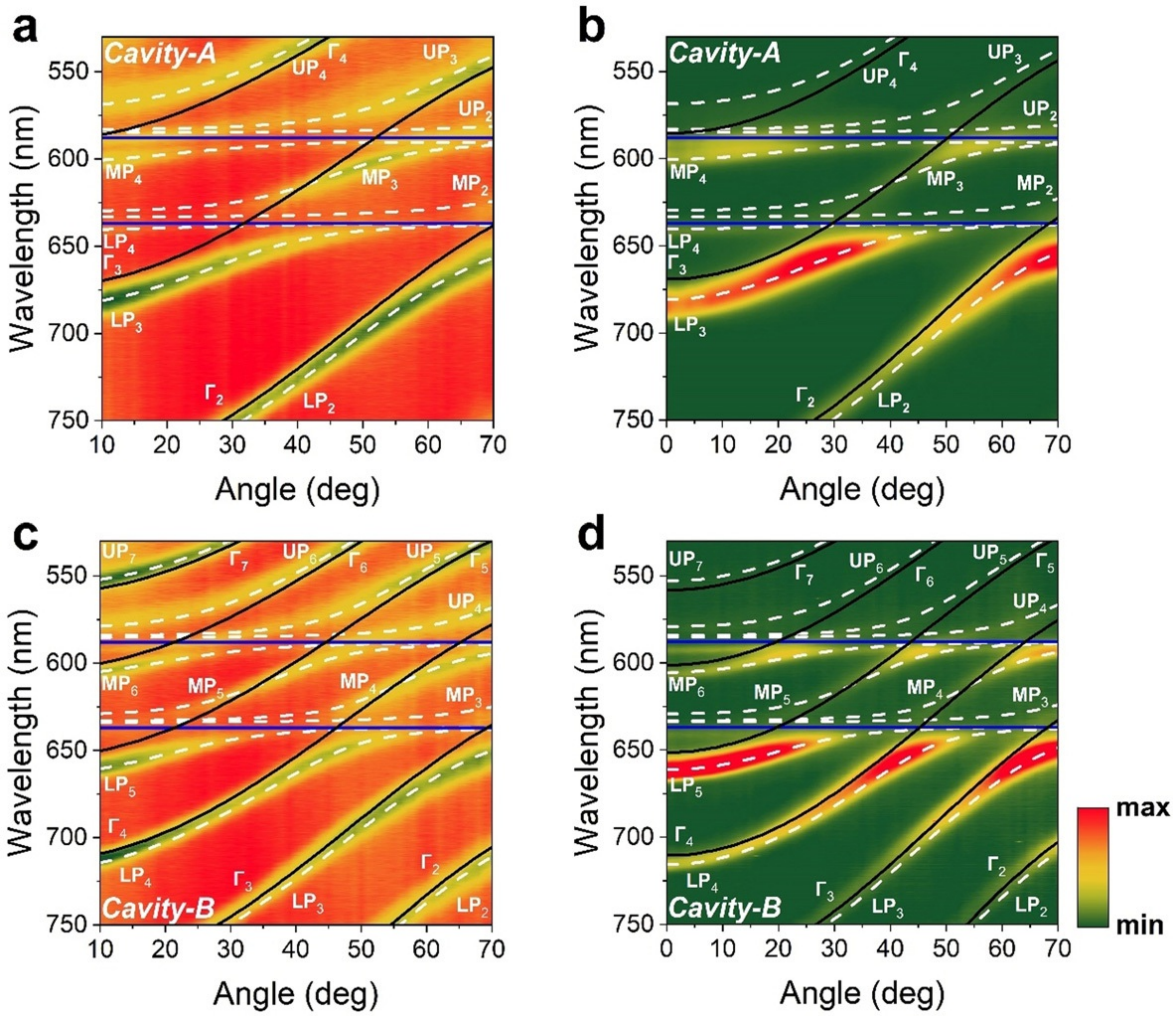
Angle‐resolved (a) white light reflectivity and (b) PL spectra from Cavity‐A containing a PS spacer layer having a thickness of 1160 nm. Part (c) shows angular white light reflectivity and (d) PL spectra from Cavity‐B that contained a 2150 nm thick PS spacer. Horizontal blue lines indicate the peak wavelength of TDBC and NK‐2707, black lines plot the different uncoupled photon modes (labelled as Γ_1,2,3…_) and white dashed lines represent the various polariton branches (labelled as LP_1,2,3…_ for lower, MP_1,2,3…_ for middle and UP_1,2,3…_ for upper branch) whose energies are calculated using a coupled oscillator model.

First, we discuss results acquired from Cavity‐A that contained a PS spacer layer having a thickness of 1160 nm, with the cavity having a total active layer thickness of 1403 nm. To demonstrate that the cavity operated within the strong coupling regime, it was characterised by angle‐resolved white light reflectivity measurements using a goniometer setup. Figure 2 a plots a white light reflectivity map recorded for angles between 10° and 70°. Figure 2 b shows angular PL emission from the same cavity when excited non‐resonantly using a CW laser diode at 405 nm, with emission collected over the angular range 0° to 70°.

It can be seen that due to the extended path length of the cavity, there are a series of different optical branches evident, that undergo an angular dependent interaction with the two excitonic states. Indeed, we have previously studied a 3 μm thick multimode microcavity filled with J‐aggregates of the dye TDBC and have shown that such structures are characterised by a ladder of polaritonic states.^[34]^ To model the optical properties of Cavity‐A, we have used an approach recently proposed in a theoretical work by Balasubrahmaniyam et al.^[35]^ and experimentally confirmed by Georgiou et al.^[36]^ The model is based on a 12‐level coupled oscillator shown in matrix Equation 1 describing the hybridisation of 4 individual photon modes with the two J‐aggregated excitons. This new model is described in more detail in the Supporting Information and also compared with another approach that was previously used to describe multimode microcavity structures (Figure S3a–d).
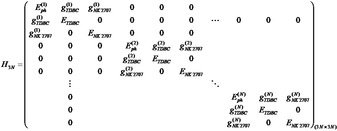



Using this model, we predict the angular dependent energy of the various polaritonic states that occur as a result of strong exciton–photon coupling. The results of our simulations are shown in Figure 2 a and b, where we overlay the measured optical reflectivity and PL emission intensity with the predicted polariton energies. Here, the blue horizontal lines represent the peak absorption wavelength of the two excitons, the black lines show the uncoupled photonic modes extracted from a TM model and the white dashed lines the predicted dispersion of the various polariton branches whose energies are calculated using a 12‐level coupled oscillator model. For the sake of simplicity, we label the uncoupled photon modes using Γ_1,2,3…_, with the various polariton branches labelled using LP_1,2,3…_ for the lower, MP_1,2,3…_ for the middle and UP_1,2,3…_ for the upper polariton‐branch states. Note that for clarity, Figure 2 a–d plots data over the spectral range 530–750 nm. For completeness however, we re‐plot the same reflectivity and PL data in Figure S4a and b of the Supporting Information where we include the whole spectral range measured during the experiment. Here, we omit simulations for branches LP_1_, MP_1_ and UP_1_, as photon mode Γ_1_ is very negatively detuned, with these middle and upper polariton branches having negligible photon component within the measured angular range (i.e. the branches are almost completely excitonic).

In Figure 2 a, it can be seen that the various polariton modes undergo an avoided crossing at wavelengths associated with the peak absorption of the two excitons; an observation consistent with a microcavity operating in the strong coupling regime. A similar effect is also shown in PL emission data of Figure 2 b. It can be seen that polariton branches MP_2_, MP_3_ and MP_4_ are located between the wavelengths characteristic of the TDBC and NK‐2707 J‐aggregate excitons. We have previously shown that strong mixing between excitons and cavity‐photons occur in such hybridised middle polariton branches.[^10^, ^11^, ^37^] We further confirm mixing and hybridisation in Supporting Information Figure S5 where we plot the relevant Hopfield coefficients for branches LP_3_, MP_3_ and UP_3_ and demonstrate substantial mixing between photon mode Γ_3_ and NK‐2707 and TDBC excitons.

We now use our model to extract the Rabi‐splitting energy of the two J‐aggregate dyes with the various cavity photons. For Cavity‐A, we determine (over the angular range 10° to 70°) a Rabi energy splitting ħ*Ω*_rabi_ between branches LP_2_–MP_2_ and LP_3_–MP_3_ of 100 meV; this splitting results from a coupling between the NK‐2707 exciton and photon modes Γ_2_ and Γ_3_ respectively. We also detect a splitting between branches MP_3_–UP_3_ and MP_4_–UP_4_ of 120 meV, corresponding to coupling between TDBC excitons and photon modes Γ_3_ and Γ_4_ respectively. We summarise the Rabi‐splitting energies between the various polariton branches and the excitons to which they are coupled in Table 1. We can use the values of the interaction potential *g* (through its relationship with the Rabi splitting energy 2*g*=ħ*Ω*_rabi_) and the half‐width at half‐maximum (HWHM) of the cavity‐photon (*γ_c_*) and exciton (*γ_x_*) linewidths to demonstrate that the cavity operates in the strong coupling regime. Here, *γ_c_* was determined using a TM model (i.e. by setting the oscillator strength of the two excitons to zero), together with experimental values of interaction potential extracted from our model (*g*_NK‐2707_=50 meV, *g*_TDBC_=60 meV). From measured HWHM homogeneous exciton linewidths $\gamma_{x}^{NK-2707}=$ 20 meV, $\gamma_{x}^{TDBC}=$ 15 meV and cavity‐photon HWHM linewidth *γ_c_* =6 meV, we find that the condition for strong coupling defined by Equation 2 is met for both materials.^[38]^

(2) $$g^{2}>\left(\gamma_{c}^{2}+\gamma_{x}^{2}\right)/2$$

**Table 1:** Peak energy (*E*) and HWHM linewidth (*γ_x_*) of NK‐2707 and TDBC excitons, Rabi splitting energy (ħ*Ω*) and cavity mode HWHM linewidths (*γ_c_*) used in the coupled oscillator model for Cavity‐A and Cavity‐B.

Cavity	*E*_NK‐2707_ [eV]	*E*_TDBC_ [eV]	${\hbar \Omega }_{Rabi}^{NK2707}$ [meV]	${\hbar \Omega }_{Rabi}^{TDBC}$ [meV]	$\gamma_{x}^{NK2707}$ [meV]	$\gamma_{x}^{TDBC}$ [meV]	*γ_c_* [meV]
A	1.95	2.11	100	120	20	15	6
B	1.95	2.11	80	90	20	15	4

We now turn our attention to Cavity‐B that contained a thicker (2150 nm) PS spacer layer placed between the two J‐aggregated dyes. As is expected, the more extended cavity length (total active layer thickness of 2393 nm) results in additional cavity‐photon modes, with the wavelength‐spacing between the modes being smaller compared to those observed in Cavity‐A. This increased number of photon modes resulted in an increased number of polariton branches. Figures 2 c and d plot 2D maps of the cavity angle‐dependent white light reflectivity and PL spectra with the uncoupled photon modes and polariton modes again labelled using Γ_1,2,3…_, LP_1,2,3…_, MP_1,2,3…_ and UP_1,2,3…_, respectively. To describe this structure, we have used a 21‐level coupled oscillator model to describe the interaction between *N*=7 photon modes and 2 excitons. We overlay the experimental data with simulations where the blue solid lines represent the peak wavelength of TDBC and NK‐2707 excitons, the black solid lines show the uncoupled cavity modes and the white dashed lines fit the various polariton branches. Again, in order to simplify Figure 2 c and d, we omit simulations of polariton branches LP_6_, LP_7_, MP_1_, MP_2_, MP_7_ and UP_1‐3_ as they are very exciton‐like (nearly non‐dispersive) over the angular range studied. Here, data is plotted over the wavelength range of 530 nm to 750 nm. For completeness, Figure S4c and d of the Supporting Information re‐plots the angle‐resolved white‐light reflectivity and PL data over the whole wavelength range measured in the experiment.

In Cavity‐B, we observe an interaction between photon modes Γ_3_, Γ_4_ and Γ_5_ with NK‐2707, with modes Γ_4_, Γ_5_ and Γ_6_ also undergoing an interaction with the TDBC excitons. We summarise the Rabi‐splitting energies that are observed between the various polariton branches in Table 1. As input into Equation (2), we again use typical fitted values of the interaction potentials of the various branches derived from our model (*g*_NK‐2707_=40 meV, *g*_TDBC_=45 meV), the HWHM linewidth of the two excitons ($\gamma_{x}^{NK-2707}=$ 20 meV, $\gamma_{x}^{TDBC}=$ 15 meV) together with the HWHM of the photon modes (*γ_c_* =4 meV) to confirm that Cavity‐B operates in the strong coupling regime.^[38]^ To illustrate the hybridisation of excitons and photons in this structure, we plot the Hopfield coefficients for polariton states LP_5_, MP_5_ and UP_5_ in Figure S6 of the Supporting Information.

Note that the linewidth of the photon modes in Cavity‐B is slightly reduced compared to those of Cavity‐A. Here, it is well known that cavity Q‐factor increases as the thickness of the active layer is increased, with this effect occurring as a result of a longer photon round‐trip propagation time.^[1]^ Indeed, our experimental white light reflectivity data has allowed us to determine the Q‐factor of Cavity‐A and Cavity‐B by finding the ratio between the centre wavelength and full‐width at half‐maximum linewidth of a negatively detuned polariton mode in each cavity (LP_2_ in Cavity‐A and LP_3_ in Cavity‐B). By doing this, we determine that Cavity‐A and Cavity‐B have Q‐factors of around 60 and 107 respectively.

**Figure 3:**
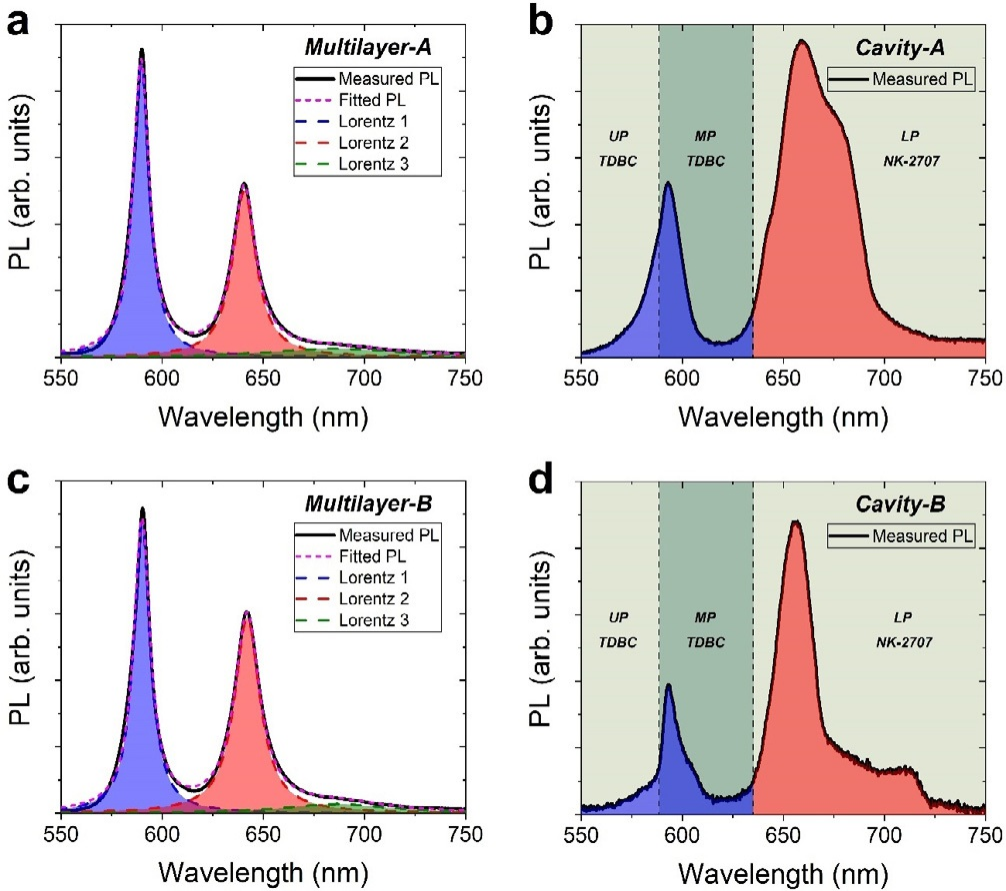
Angle‐integrated PL of a) Multilayer‐A, b) Cavity‐A, c) Multilayer‐B and d) Cavity‐B, when excited at 405 nm using a CW laser diode. In parts (a) and (c) we fit the PL with 3 Lorentz curves where blue represents PL from TDBC and red and green represent PL from NK‐2707. A convolution of the 3 Lorentz curves is shown in magenta. Parts (b) and (d) show shaded areas in blue and red that we associate with polariton PL emission from TDBC and NK‐2707 respectively. The green shaded areas mark the wavelength range of the various LPB, MPB and UPB given by our coupled oscillator model. The dashed vertical lines represent the peak wavelength of the two excitons.

We now explore energy transfer processes in both microcavities and thin‐film controls. Figure 3 a–d, shows the angle‐integrated PL emitted by Multilayer‐A, Cavity‐A, Multilayer‐B and Cavity‐B respectively for collection angles between 0° and 70°. In Figure 3 a and c, we fit the PL emission from the two multilayer films with 3 Lorentz curves (blue, red and green) with the magenta dashed line representing the sum of the 3 Lorentz curves. Excitation of the multilayer films was performed using a 450 nm laser diode. Here, we estimate that the dye layers in the films absorb an equal amount of incident laser power of around 1 %. It can be seen that the description of the experimental PL data (shown using a black solid line) is excellent. The emission associated with TDBC is shown in blue (Lorentz 1), while red and green dashed lines and shaded areas (Lorentz 2 and 3) represent PL emission from NK‐2707. By comparing the integrated areas under Lorentz curves 1, 2 and 3 we estimate that in Multilayer‐A, TDBC and NK‐2707 contribute equally to the PL emission (50–50 %). For Multilayer‐B, we find that the fraction of emission that occurs from TDBC and NK‐2707 is 46 and 54 %, respectively. We believe any differences in the emission distribution between Multilayer‐A and Multilayer‐B most likely come from slight unintended thickness variations in the different J‐aggregate layers.

We now discuss polariton PL from Cavity‐A and Cavity‐B. This is shown in Figures 3 b and d where we plot angle‐integrated PL emission from Cavity‐A and Cavity‐B recorded using a goniometer setup over collection angles between 0 and 70°. It can be seen that the emission pattern from the microcavities is different compared to their respective non‐cavity controls (Figure 3 a and c), indicating that there is a redistribution of energy within the strongly coupled cavities. To characterise the extent of the assumed energy transfer, we identify polariton branches LP_1‐4_ for *Cavity A*, and LP_1‐7_ for *Cavity B* as being states that are principally composed of a cavity photon mode and NK‐2707 excitons. From our measurement of the dispersion of such polariton branches in Figure 2, we identify the wavelength‐range of polariton states that are principally associated with NK‐2707 excitons (see shaded bands in Figure 3 b and d). By integrating under the emission curves shown in Figure 3 b and d, we find that for both Cavity‐A and Cavity‐B around 80 % of emission comes from states associated with NK‐2707 excitons (LP states), with the remainder of emission associated with polaritonic states that contain a substantial fraction of TDBC emission. These findings are consistent with previous reports on energy transfer in cavities that utilised a spacer layer having a thickness of 100 nm.^[13]^

For completeness, we performed a comparison between multilayer and cavity emission where we converted emission intensity into relative number of photons. To achieve this, the PL emission intensity *I*_PL_(*E*_PL_) at each given energy was divided by the photon energy *E*_PL_. In Figure S7 of the Supporting Information we replot data from such analysis where we find very similar results to those shown in Figure 3. Indeed, we find that emission from multilayers results equally from TDBC and NK‐2707 excitons while in both cavities around 80 % of emission is emitted by lower‐branch polariton states that are dominated by mixing between cavity photon and NK‐2707 excitons.

The redistribution of emission that occurs in the cavities as compared to that emitted by the relevant control films is also evident in Figure S8 of the Supporting Information, where we plot PL from Cavity‐A and Multilayer‐A collected at an angle of 43°. This angle corresponds to maximum hybridisation between the two excitons and has been identified based on the Hopfield coefficients for states in polariton branch MP_3_ as shown in Figure S5 of the Supporting Information.

Our simple analysis therefore indicates that there is a substantial redistribution of energy from states associated with the higher‐energy TDBC states to lower‐energy states associated with NK‐2707. This energy transfer process occurs via the various hybridised middle‐branch polariton modes which are characterised by a high degree of mixing between the two excitonic species and one or more cavity photon modes (see Figure S5 and S6 of the Supporting Information).^[11]^ These MP states act as a new non‐radiative relaxation pathway between the two exciton reservoirs that, as we show, is effective even when the molecules are separated by a micron‐thick spacer layer.^[11]^ Here, we have previously shown that excitons in the higher‐energy TDBC reservoir are able to populate (scatter) into lower lying MP states. These polaritons can then in turn decay non‐radiatively into the lower‐lying NK‐2707 reservoir, resulting in an energy‐transfer process. Although reasonably effective, this energy transfer process appears incomplete as confirmed by emission from higher energy states that have a significant TDBC exciton fraction. Note however that we include hybrid middle polariton branches that contain both NK‐2707 and TDBC states (MP_1‐4_ in Cavity‐A and MP_1‐7_ in Cavity‐B) as states that are dominated by TDBC. This arbitrary assignment will likely result in an under‐estimate of the efficiency of energy transfer.

We can also estimate the efficiency of energy transfer by determining the relative number of polaritons along each polariton branch. This analysis accounts for the fact that photon‐like polariton states are more emissive than exciton‐like states. Here, we use the Hopfield coefficients calculated for each polariton branch as input into Equation 3 which converts the angle‐dependent polariton luminescence intensity *I*(*θ*) to polariton population density *f_k_* (*θ*) along each polariton branch of Cavity‐A and Cavity‐B.^[37]^

(3) $$f_{k}\left(\theta \right)=\frac{I\left(\theta \right)}{{{|\alpha }_{\gamma }\left(\theta \right)|}^{2}\;{{|E}_{p}\left(\theta \right)|}^{2}\cos\theta \;}$$

Here, *E_p_* (*θ*) is the energy of the polariton modes and *α*_*γ*_(*θ*) is the photon fraction of the branch at any given angle. We can then compare the angle‐integrated number of polaritons along a particular polariton branch with the total polariton population integrated over all branches (for more details regarding this calculation see Ref. [37]) to get an additional measure of the efficiency of polariton energy transfer. Following such analysis, we find that the polariton population associated with NK‐2707 excitons‐polaritons is 62 % in Cavity‐A (integrated along branches LP_1‐4_ and including states in branches MP_2‐4_ associated with NK‐2707 excitons as indicated by the Hopfield coefficients), and 68 % in Cavity‐B (integrated along branches LP_1–7_ and including states in branches MP_3–6_ associated with NK‐2707 excitons as indicated by the Hopfield coefficients). This analysis again indicates a significant fraction of energy transfer between states associated with TDBC to states associated with NK‐2707.

We have previously shown that efficient energy transfer between exciton states in a hybrid semiconductor microcavity containing either a significantly thinner spacer layer or a blended active layer was mediated by the hybridised MP states.[^11^, ^37^] To determine whether such a process also underpins energy transfer in the “micron‐thick” multilayer cavities studied here, we have performed PLE spectroscopy to determine the efficiency of this relaxation process. Here, measurements were performed on Cavity‐A, with the structure excited as a function of angle (between 14 and 70°) and as a function of wavelength (between 500 and 660 nm) while detecting the intensity of PL emission at 0° and 685 nm. This measurement effectively allowed us to map the efficiency of relaxation from any state in the cavity dispersion plot to the bottom of polariton branch LP_3_. Note that we have corrected the PLE data at each point for the amount of laser absorbed within the active layer of the cavity. Furthermore, we have also applied a correction to account for excitation light that was absorbed by the metallic cavity mirrors, with this relative absorption determined using a TM model.

**Figure 4:**
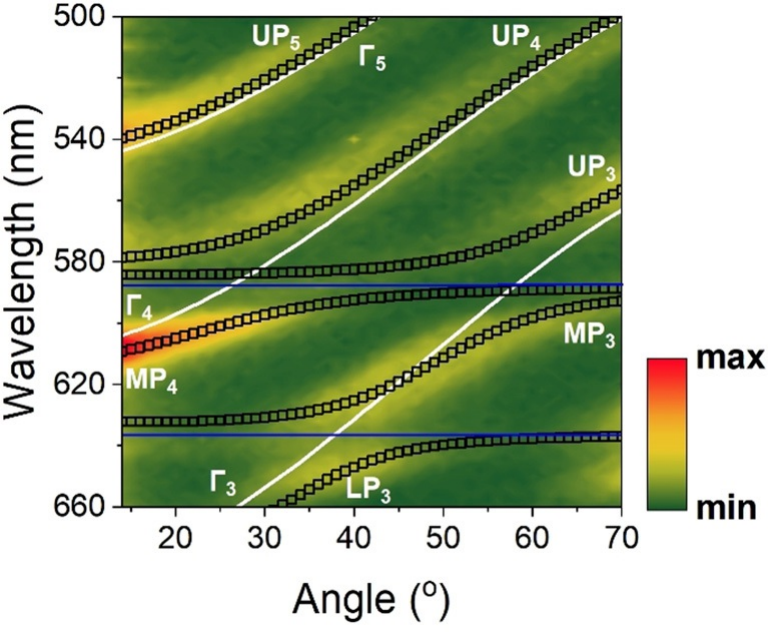
PLE measurement of Cavity‐A when excited between 500 nm and 660 nm over the angular range 14° to 70° and collected at 0° at a wavelength of 685 nm which coincides with the bottom of the polariton branch LP_3_. Horizontal blue lines indicate the peak wavelength of TDBC and NK‐2707, white lines plot the different uncoupled photon modes (labelled as Γ_1,2,3…_) and black open squares represent the various polariton branches (labelled as LP_1,2,3…_ for lower, MP_1,2,3…_ for middle and UP_1,2,3…_ for upper branch) whose energies are calculated using a coupled oscillator model.

Here, typical results are plotted in Figure 4, which shows a 2D plot of the PLE efficiency when detecting emission from the bottom of polariton branch LP_3_. Here, we overlay the experimental data with a coupled oscillator model. The white solid lines represent the uncoupled photon modes, the black open squares correspond to the various polariton states and the peak absorption energy of the TDBC and NK‐2707 excitons are marked using blue solid lines. Note that we have performed measurements at a slightly different place on Cavity‐A surface as compared to that reported in Figure 2 a, with excitons undergoing resonance with the photon modes at slightly different angles due to a small, unintended thickness variation of the various layers. Here, polariton branch MP_4_ appears particularly “bright”, indicating that it acts as an efficient energy transfer pathway to the bottom of branch LP_3_. This finding is in accord with our previous measurements that have demonstrated that TDBC excitons are able to scatter into such hybrid polariton states and then undergo relaxation to the NK‐2707 reservoir, from where excitons then scatter into polariton states at lower energy.^[11]^

## Conclusion

Microcavities were fabricated containing strongly‐coupled J‐aggregates that were separated by a spacer layer having a thickness of up to 2 μm. By comparing angular‐dependent cavity emission with emission from non‐cavity control structures, it was demonstrated that energy transfer occurs with reasonable efficiency (between 60 and 80 %) between the different molecular dyes within the microcavities that is mediated by hybrid polariton states. Photoluminescence excitation spectroscopy measurements are then used to confirm that hybrid polariton states, composed of a mixture between the cavity photon modes and the different exciton states, act as a route for efficient ultralong‐range energy transfer to energetically lower‐lying states. We believe that the use of hybrid polariton states to transfer energy over mesoscopic (micron‐length) distances will be of significant potential interest in a range of technologies, including solar cells and microfluidic devices that exploit polaritonic effects to modify chemical reactions.

## Conflict of interest

The authors declare no conflict of interest.

## Supporting information

As a service to our authors and readers, this journal provides supporting information supplied by the authors. Such materials are peer reviewed and may be re‐organized for online delivery, but are not copy‐edited or typeset. Technical support issues arising from supporting information (other than missing files) should be addressed to the authors.

Supplementary
