# Supplementary material for: Ultralong‐Range Polariton‐Assisted Energy Transfer in Organic Microcavities
Source: Angew Chem Int Ed Engl. 2021 Jun 18;60(30):16661–7. doi: 10.1002/anie.202105442 (PMC8361947; doi:10.1002/anie.202105442)
Supplement: Supplementary file 1 — Supplementary [file ANIE-60-16661-s001.pdf]

Angewandte Chemie
Eine Zeitschrift der Gesellschaft Deutscher Chemiker

Supporting Information

Ultralong-Range Polariton-Assisted Energy Transfer in Organic Microcavities

Kyriacos Georgiou, Rahul Jayaprakash, Andreas Othonos, and David G. Lidzey**

anie_202105442_sm_miscellaneous_information.pdf

Author Contributions

D.G.L. conceived the idea. K.G. designed and fabricated the samples. K.G. designed and performed the steady state optical experiments. R.J. performed the TM and coupled oscillator simulations. K.G. and A.O. conducted pump-probe characterisation measurements. K.G. analysed the data. All the authors discussed the results. K.G. wrote the manuscript with significant contributions from D.G.L. who also oversaw the whole project.

Table of Contents

1. **Film femtosecond pump-probe spectroscopy and PLE measurements** S2
2. **Conventional (*N*+2) Hamiltonian model Vs. New 3*N* Hamiltonian model** S3
3. **Hopfield coefficients** S7
4. **Multilayer Vs. Cavity photon emission distribution** S8
5. **Materials and Methods**... S10
6. **References** S11

1. Film femtosecond pump-probe spectroscopy and PLE measurements

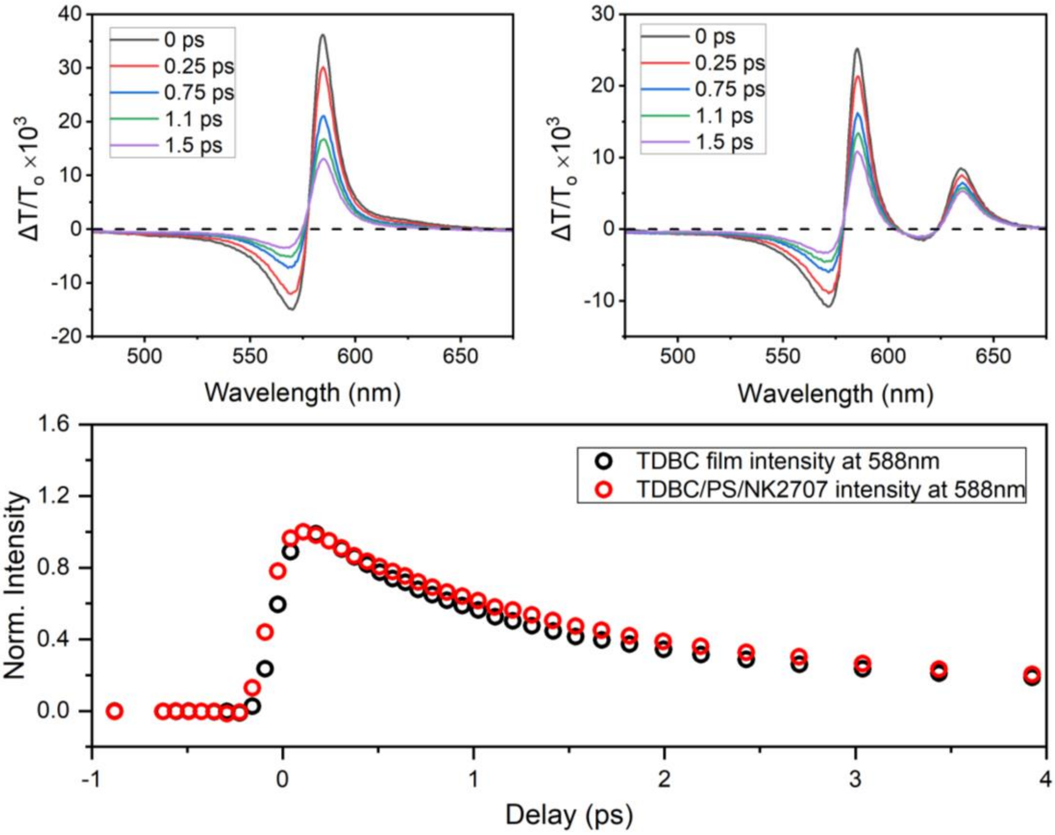

Figure S1. Top Left: Pump-probe measurement showing the differential transmission $\Delta T/T_o$ of a TDBC / PS film for various delay times between pump and probe. Top Right: Pump-probe measurement showing the differential transmission $\Delta T/T_o$ of a TDBC / PS / NK-2707 multilayer film for various delay times between pump and probe. Bottom: Normalised intensity of the differential transmission at 588 nm for a range of delay times.

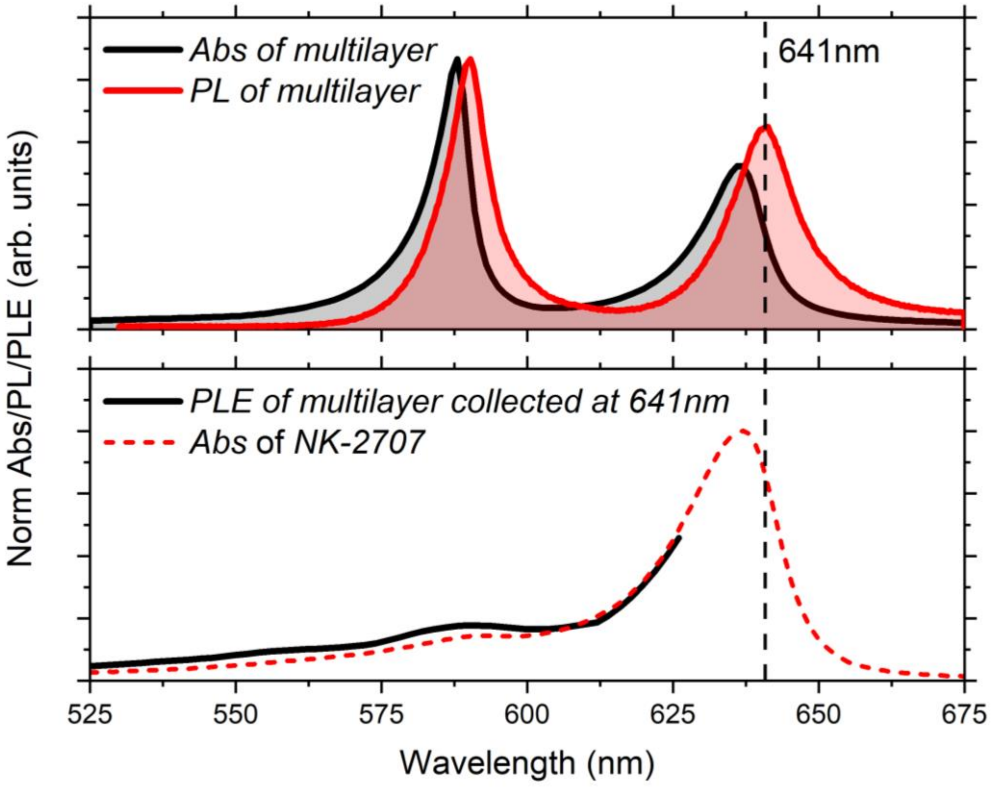

Figure S2. Top: Absorption and PL spectra of a multilayer film of TDBC / PS / NK-2707. Bottom: PLE measurement of the same film (black line) when excited between 525 nm and 625 nm and collected at a wavelength of 641 nm which coincides with the emission peak of NK-2707 as shown by the vertical dashed line. For comparison, the red dashed curve shows the absorption spectrum of a NK-2707 film.

2. Conventional (*N*+2) Hamiltonian model Vs. New 3*N* Hamiltonian model

Our approach, recently proposed in a theoretical work by Balasubrahmaniyam *et al.*[1] and experimentally confirmed by Georgiou *et al.*[2] is different from the (N+1) x (N+1) Hamiltonian that we have used previously to describe multimode optical cavities[3]. Here, the model is based on a 3N x 3N Hamiltonian shown in matrix Equation (1) of the manuscript, where there are 3 individual species that can mutually hybridise (namely photon [E_{ph}], TDBC exciton [E_{TDBC}] and NK-2707 exciton [E_{NK2707}]) with *N* being the number of dispersive optical modes that become resonant with the two excitons. The model implicitly assumes that the system transitions to a new regime within strong coupling where the various optical modes are energetically decoupled from each other, with the excitons interacting with each optical mode independently[1].

We show in Figure S3a-d that the use of this 3N x 3N Hamiltonian results in an improved fit compared to a model based on a conventional (N+2) x (N+2) Hamiltonian in which simultaneous interactions occur between the two excitons and all N optical states. Specifically, we plot the same experimental reflectivity maps shown in the paper (Figure 2a and c) however we plot data over a reduced spectral range (550 – 700 nm) and overlay it with the polariton energies extracted from the two different models. This is shown in Figure S3a and c, where we overlay data with a photon-decoupled 3*N* Hamiltonian model described in Equation (1), while Figure S3b and d shows the same data that is instead modelled using a conventional *N*+2 Hamiltonian described in Equation (S1). Note that we have used the same interaction potential values (g) and dispersive optical modes $\Gamma_{1,2,3\ldots}$ in both models.

Although we find that the "conventional" *N*+2 Hamiltonian model shown in Figure S3b and d fits the experimental data relatively well, we observe a small deviation between experimental and simulation polariton modes, particularly at energetic regions around the exciton energies. For instance, in Figure S3b, polariton mode P_5 crosses the TDBC exciton (vertical white dashed line) at a wavelength of 588 nm and an angle of 35°. At this point, it is apparent from the experimental data that there is a splitting of the polariton mode which is only correctly described by the 3*N* Hamiltonian model used in Figure S3a (splitting between modes MP_4 and UP_3). This deviation between the experimental data and the *N*+2 Hamiltonian model of Equation (S1) can also be seen for polariton modes $P_{4\text{-}7}$ in Figure S3d at the points where the polariton modes cross the energy of the NK-2707 and TDBC excitons (white horizontal dashed lines). In all cases the new model shown in Figure S3c describes the mode splitting observed in the experimental data resulting in an improved fit.

$$H_{N+2} = \begin{pmatrix} E_{TDBC} & 0 & g_{TDBC}^{(1)} & g_{TDBC}^{(2)} & & g_{TDBC}^{(N)} \\ 0 & E_{NK2707} & g_{NK2707}^{(1)} & g_{NK2707}^{(2)} & \cdots & g_{NK2707}^{(N)} \\ g_{TDBC}^{(1)} & g_{NK2707}^{(1)} & E_{ph}^{(1)} & 0 & & 0 \\ g_{TDBC}^{(2)} & g_{NK2707}^{(2)} & 0 & E_{ph}^{(2)} & & 0 \\ & \vdots & & & \ddots & 0 \\ g_{TDBC}^{(N)} & g_{NK2707}^{(N)} & 0 & 0 & 0 & E_{ph}^{(N)} \end{pmatrix}_{(N+2)\times(N+2)} \qquad \text{(S1)}$$

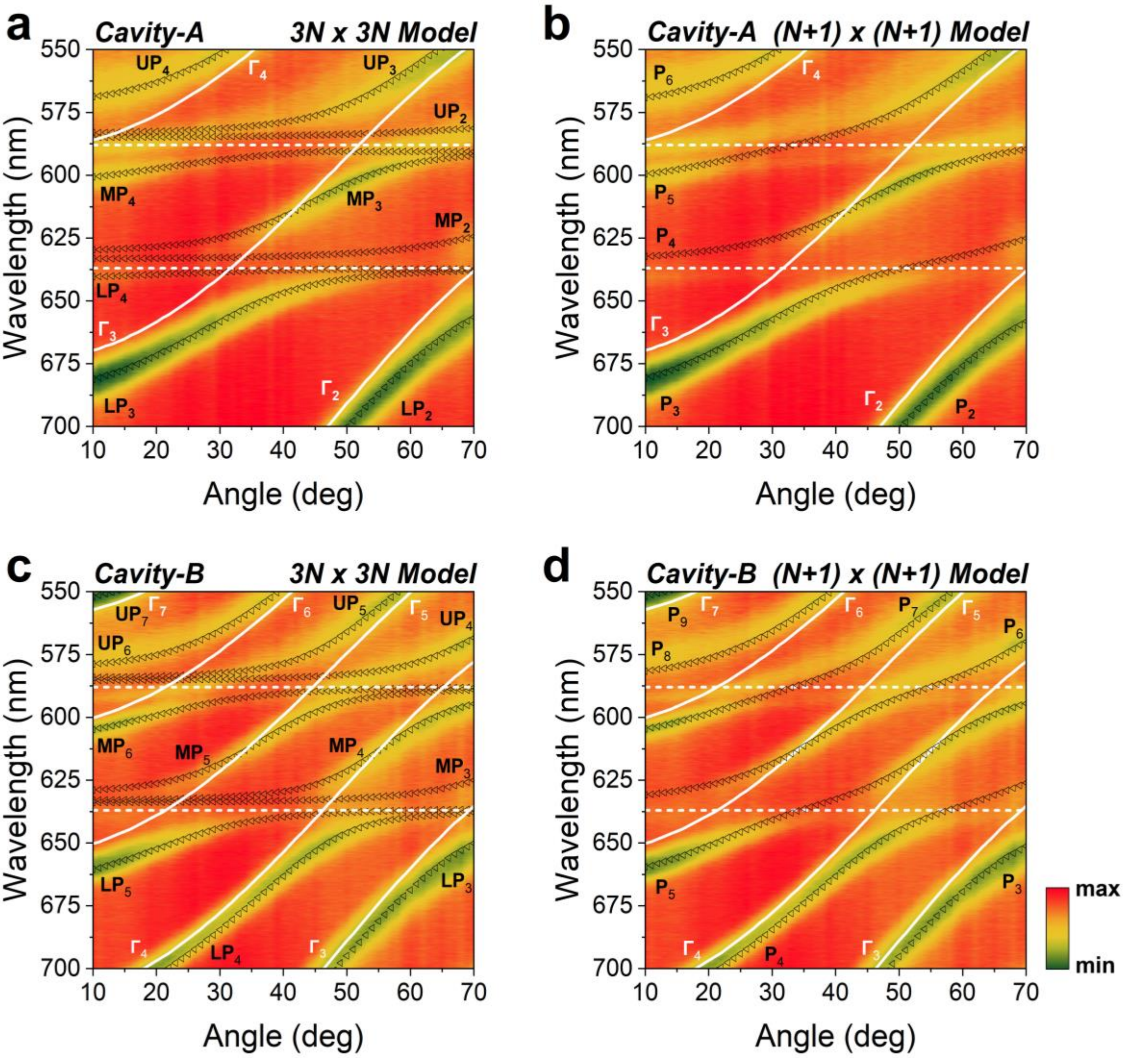

Figure S3. Angle-resolved white light reflectivity maps of *Cavity A* and *Cavity B* fitted with (a and c) a decoupled 3*N* Hamiltonian model and (b and d) a conventional *N*+2 Hamiltonian model. The white dashed lines indicate the peak absorption wavelength of the TDBC and NK-2707. The uncoupled optical modes are shown with white solid lines and labelled as $\Gamma_{1,2,3\ldots}$. The polariton modes are marked with open triangles and labelled as $LP_{1,2,3\ldots}$, $MP_{1,2,3\ldots}$, $UP_{1,2,3\ldots}$ and $P_{1,2,3\ldots}$.

For completeness, in Figure S4a-d we also re-plot the angle-resolved white-light reflectivity and PL data shown in Figure 2 of the manuscript. Here we plot the figure over a more extended wavelength range (between 530 nm to 805 nm), allowing photon mode Γ_1 and polariton branch LP_1 to be seen.

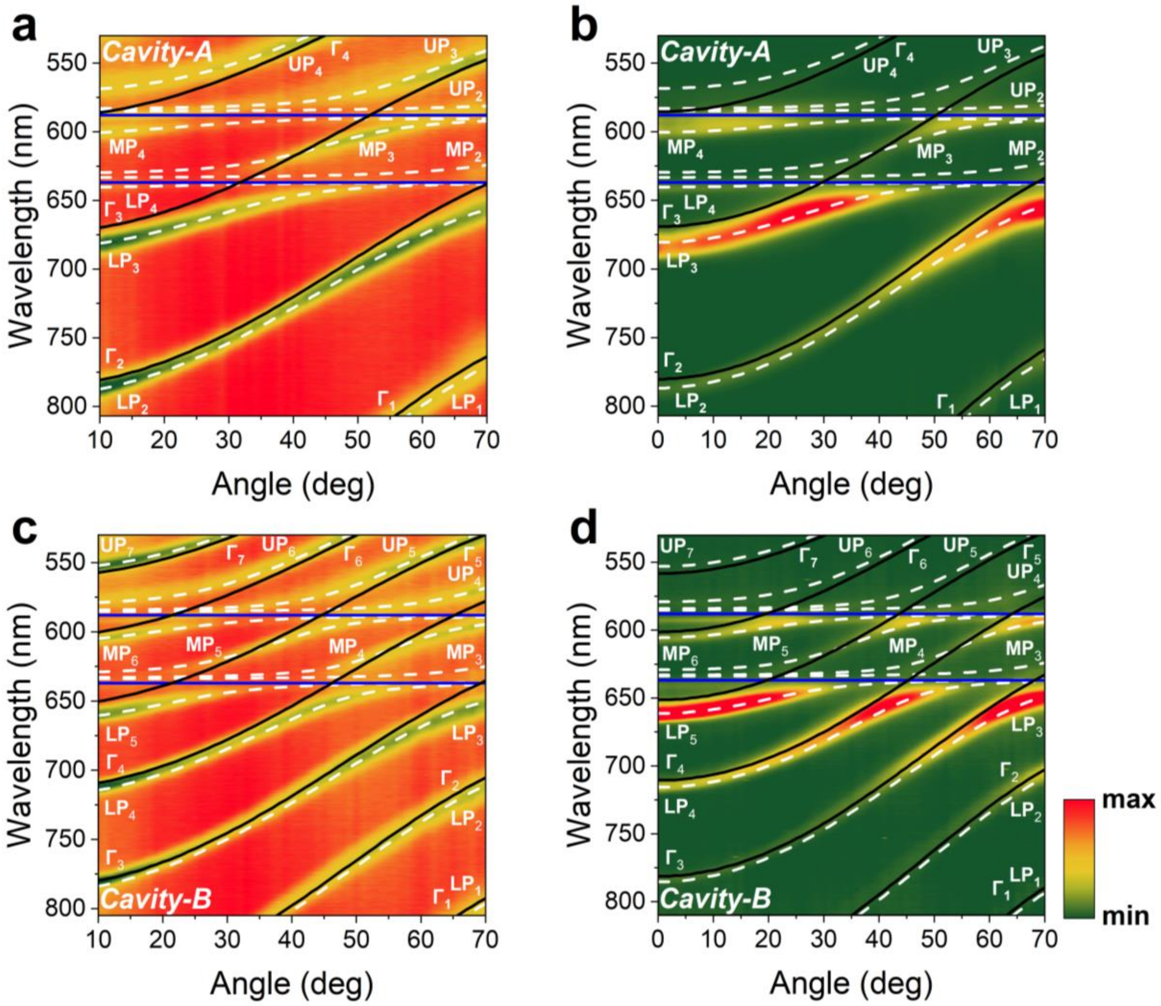

Figure S4. Angle-resolved (a) white light reflectivity and (b) PL spectra from Cavity-A containing a PS spacer layer having a thickness of 1160 nm. Part (c) shows angular white light reflectivity and (d) PL spectra from Cavity-B that contained a 2150 nm thick PS spacer. Horizontal blue lines indicate the peak wavelength of TDBC and NK-2707, black lines plot the different uncoupled photon modes (labelled as $\Gamma_{1,2,3\ldots}$) and white dashed lines represent the various polariton branches (labelled as LP1,2,3… for lower, $MP_{1,2,3\ldots}$ for middle and $UP_{1,2,3\ldots}$ for upper branch) whose energies are calculated using a coupled oscillator model.

3. Hopfield coefficients

Figure S5 plots the Hopfield coefficients for LP_3, MP_3 and UP_3 of *Cavity-A*. As it can be seen, middle branch MP_3 is a mixture of the Γ_3 photon-mode, with NK-2707 and TDBC excitons, with mixing being maximised at an angle of ~43°. Figure S6 shows the Hopfield coefficients of *Cavity-B* for polariton branches LP_5, MP_5 and UP_5 with MP_5. Here a high degree of mixing is observed between photon-mode Γ_5 and NK-2707 and TDBC excitons at an angle of ~35°.

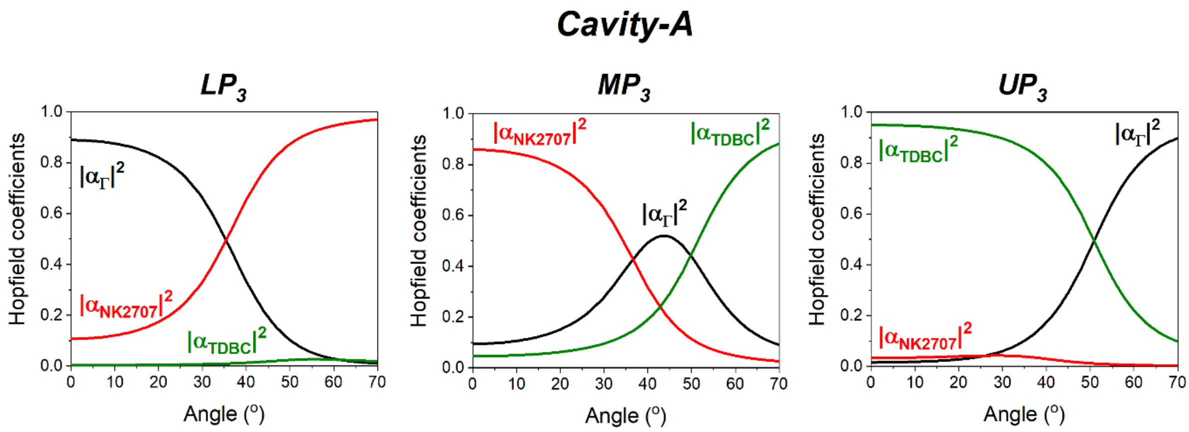

Figure S5. Hopfield coefficients showing the mixing fractions of photon mode Γ_3 and excitons NK-2707 and TDBC in polariton branches LP_3 (left), MP_3 (centre) and UP_3 (right) of *Cavity A*.

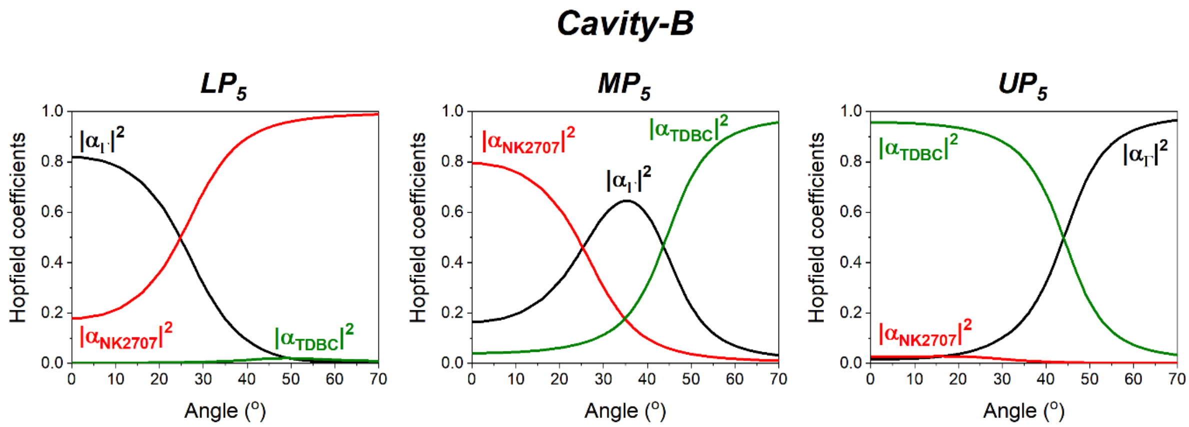

Figure S6. Hopfield coefficients showing the mixing fractions of photon mode Γ_5 and excitons NK-2707 and TDBC in polariton branches LP_5 (left), MP_5 (centre) and UP_5 (right) of *Cavity B*.

4. Multilayer Vs. Cavity photon emission distribution

Figure S7 plots the relative number of photons emitted from *Multilayer-A*, *Cavity-A*, *Multilayer-B* and *Cavity-B*. To convert PL intensity to relative number of photons we have divided it by the energy of photons emitted. In *Multilayer-A* emission originated equally from TDBC and NK-2707 excitons (50%-50%). In *Multilayer-B* TDBC excitons contribute to 49% of emission while 51% of emission is associated with NK-2707 excitons. In *Cavity-A* 77% of emission result from LP states while the remaining 23% of photons come from MP and UP states. In *Cavity-B* 76% of emission originates from LP states and 24% from MP and UP states.

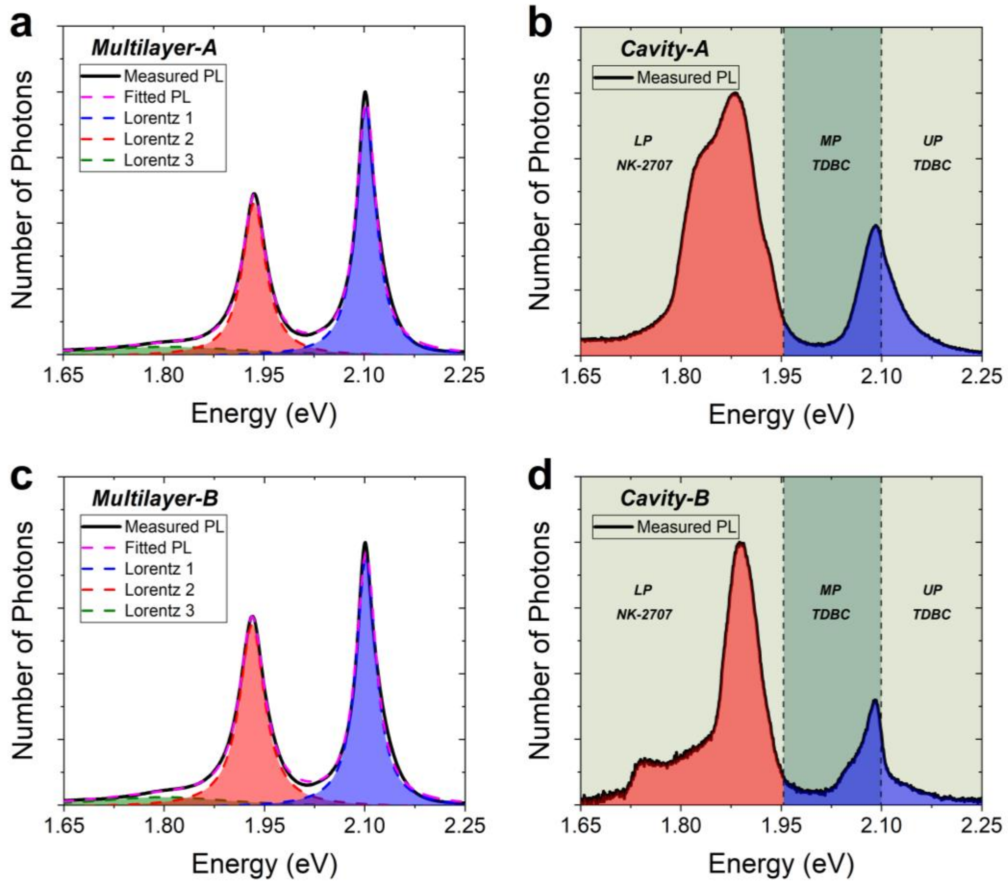

Figure S7. Relative number of angle-integrated photons emitted by (a) *Multilayer-A*, (b) *Cavity-A*, (c) *Multilayer-B* and (d) *Cavity-B*. In parts (a) and (c) we fit the number of photons with 3 Lorentz curves where blue represents photons emitted by TDBC and red and green represent photons emitted by NK-2707. A convolution of the 3 Lorentz curves is shown in magenta. Parts (b) and (d) show shaded areas in blue and red associating polariton photon emission to TDBC and NK-2707 respectively. The green shaded areas mark the energy range of the various LP, MP and UP states given by our coupled oscillator model. The dashed vertical lines represent the peak wavelength of the two excitons.

In Figure S8 we plot a cross-section of PL emission from *Cavity-A* at an angle corresponding to a maximum mixing between photons and the two excitons at θ = 43°, along with PL emission from the *Multilayer-A* control film that was also collected at the same angle. Here, it can be clearly seen that at this angle of maximum hybridisation between the two excitons, a redistribution of energy occurs between the molecular donor species to the acceptor species which is positioned at lower energy. Here the angle of maximum mixing (θ = 43°) was identified using the Hopfield coefficients plotted in Figure S5 of the Supporting Information.

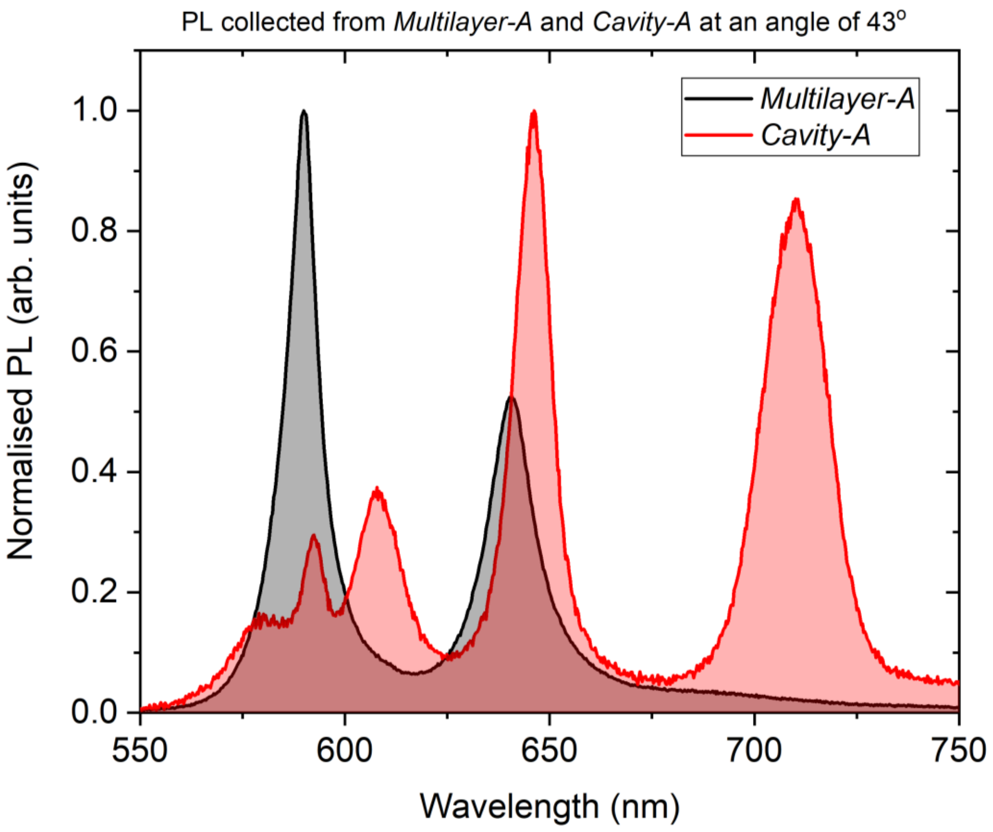

Figure S8. PL data from *Multilayer-A* (black) and *Cavity-A* (red). PL was collected at an angle of 43° for both the cavity and the film. This angle corresponds to a maximum hybridisation between the two excitons in *Cavity-A*.

5. Materials and Methods

Organic molecule solutions and films. TDBC (supplied by FEW Chemicals GmbH) and NK-2707 (supplied by Hayashibara Biochemical) were dissolved at 10% and 5% by mass in a DI water / gelatine solution (20 mg mL^{-1}), respectively. Films were spin-coated from 100 µL of the solution held at a temperature of 65°C. PS (supplied by Sigma-Aldrich) of molecular weight M_w ~ 350,000 was dissolved in toluene at 100 mg mL^{-1} and spin-coated using 200 µL of solution held at room temperature. The thickness of the various layers was controlled by changing the rotation speed of the substrate during spin-coating and was determined using a Bruker Dektak XT profilometer.

Microcavity fabrication. Ag mirrors were evaporated using an Ångstrom Engineering thermal evaporator. The sample chamber was held at a base pressure of 2×10^{-6} mbar and the deposition rate was kept between 0.5 and 1 Å/s^{-1}. The bottom mirror had a thickness of 200 nm while the top mirror was semi-transparent having a thickness of 34 nm. The organic multilayers were spin-coated as described above from J-aggregate / gelatine and PS solutions.

Angle-resolved white light reflectivity and PL. Angle-dependent white light reflectivity measurements were performed using a goniometer setup consisting of two arms attached to a motorised rotation stage. A fibre-coupled Halogen-Deuterium white light source (DH-2000-BAL) was attached to the first arm and light was focused on the sample using a series of lenses. Reflected light was collected through a series of lenses mounted on the second arm and directed into an Andor Shamrock SR-303i-A CCD spectrometer using an optical fibre. For angle-resolved PL measurements, samples were excited close to normal incidence using a 405 nm CW laser diode. PL was collected through the same motorised arm used to collect light in reflectivity measurements.

PLE measurements. Laser excitation was performed using a Fianium Supercontinuum laser with 6 ps pulse and 40 MHz repetition rate. Broadband laser light was filtered through a SPEX 270M monochromator to tune the excitation wavelength. The same goniometer setup described above was used for the excitation of the sample at different angles, with two photodiodes (SM1PD1A) added in the excitation and collection paths to measure the intensity of the incident and reflected excitation light. PL from the sample was collected at normal

incidence using lenses mounted on a third arm and then directed into an Andor Shamrock SR-303i-A CCD spectrometer.

Femtosecond pump-probe measurements. Excitation of the sample was performed using a 400 nm frequency-doubled Ti:Sapphire laser amplifier having a 1 kHz repetition rate and a pulse width of 100 fs. The white-light continuum probe was generated using a few microjoules of the amplified pulse at 800nm which was focused on a sapphire glass plate. Different delays were achieved using a retroreflector configuration mounted onto a motorised stage. The femtosecond pump-probe experiment was performed using a typical non-collinear setup where the excitation beam was directed onto the sample close to normal incidence while the probe beam was incident onto the sample at an angle of ~5°. The signal was either collected through a fibre bundle and then imaged into a spectrometer, or filtered through a 10 nm band-pass filter and then sent to a lock-in amplifier.

6. References

[1] M. Balasubrahmaniyam, C. Genet, T. Schwartz, *arXiv:2005.03527* **2020**.

[2] K. Georgiou, K. E. McGhee, R. Jayaprakash, D. G. Lidzey, *J. Chem. Phys.* **2021**, *154*, 124309.

[3] D. M. Coles, D. G. Lidzey, *Appl. Phys. Lett.* **2014**, *104*, 191108.
